# Genital Abnormalities and Growth Retardation as Early Signs of Dilated Cardiomyopathy with Ataxia Syndrome

**DOI:** 10.1155/2024/8860889

**Published:** 2024-01-20

**Authors:** Kyriaki Papadopoulou-Legbelou, Maria Ntoumpara, Maria Kavga, Eleni P. Kotanidou, Ioannis Papoulidis, Assimina Galli-Tsinopoulou, Maria Fotoulaki

**Affiliations:** ^1^4^th^ Department of Pediatrics, School of Medicine, Faculty of Health Sciences, Aristotle University of Thessaloniki, “Papageorgiou” General Hospital, Thessaloniki, Greece; ^2^Unit of Pediatric Endocrinology and Metabolism, 2^nd^ Department of Pediatrics, School of Medicine, Faculty of Health Sciences, Aristotle University of Thessaloniki, AHEPA University General Hospital, Thessaloniki, Greece; ^3^Access to Genome P.C, Clinical Laboratory Genetics, Ethnikis Antistasis 33A, Thessaloniki 55133, Greece

## Abstract

Dilated cardiomyopathy with ataxia syndrome is a rare mitochondrial disease caused by autosomal recessive mutations in the *DNAJC19* gene. The disease has been described in detail in the Canadian Hutterite population, but a few sporadic cases with de novo mutations have been published worldwide. We describe a homozygous pathogenic variant in the *DNAJC19* gene, diagnosed in Northern Greece, presenting with genital anomalies, growth failure, cardiomyopathy, and ataxia, but without increased urinary 3-methylglutaconic acid and additional presence of vitamin D disorders, hypercalciuria, and osteopenia. This case not only expands the clinical characteristics of 3-methylglutaconic aciduria type V (MGCA5) but also highlights the power of genetic analysis for detecting a diagnosis when the metabolic screen is negative.

## 1. Introduction

Dilated cardiomyopathy with ataxia syndrome (DCMA syndrome), also known as 3-methylglutaconic aciduria type V (MGCA5), is a rare mitochondrial disease caused by autosomal recessive mutations in the *DNAJC19* gene [1]. The disease has been described in detail in the Canadian Hutterite population of Southern Alberta [1]. Since then, a few sporadic cases with de novo mutations have been published worldwide [2–5].

Clinical features of MGCA5 disorder are heterogeneous. The main characteristics are dilated cardiomyopathy with nonprogressive cerebellar ataxia. However, there are other clinical characteristics, which may raise suspicion for diagnosis, such as intrauterine growth retardation, growth failure (after birth), male genital anomalies, and increased biochemical markers of mitochondrial dysfunction in plasma and urine [6]. In addition, features such as microcytic anemia, hepatic steatosis, mental retardation, and optic nerve atrophy have been described as well [4]. In the first report in Europe, which was in the Finnish population, there was a combination of dilated cardiomyopathy with noncompaction cardiomyopathy [2].

We describe a pathogenic variant (MGCA5#610198) in the *DNAJC19* gene, diagnosed in Northern Greece, which expands the phenotypic findings of this rare condition [7].

## 2. Case Report

A 5.5-year-old boy, the first child of two healthy nonconsanguineous parents, was born full-term with a birth weight of 2.550 g (10^th^ percentile) and a height of 47.5 cm (50^th^ percentile). The infant's mother experienced two previous spontaneous abortions. The neonate presented with genital abnormalities, including bilateral cryptorchidism, a small hypoplastic scrotum, and an extreme microphallus.

An endocrinology evaluation was performed during the first days of life in order to differentiate the underlying disorder of sexual development according to guidelines [8]. After the molecular confirmation of a male karyotype (46,XY), the presence of disorder of gonadal development was confirmed with complete gonadal dysgenesis (bilateral testicular dysgenesis). The presence of two extremely small, dysfunctional dysgonadal testes, in an intra-abdominal position, was confirmed after magnetic resonance imaging, whereas plasma steroids profiling confirmed low levels of testosterone for age and chromosomal gender of the offspring. Disorders of androgen synthesis and syndromic forms of androgen synthesis (such as Smith–Lemli–Opitz syndrome) or disorders associated with congenital adrenal dysfunction and androgen insensitivity syndromes were ruled out. The rest endocrinology evaluation revealed the normal function of the hypothalamus-pituitary axis with mild elevation of gonadotropins due to the absence of negative feedback loop, normal adrenal function, and euthyroidism.

At the age of 6 months, his weight dropped below the 3^rd^ percentile and his height to the 25^th^ percentile. Furthermore, he had increased liver enzymes: aspartate aminotransferase (AST): 120 U/L (normal < 48 U/L), alanine aminotransferase (ALT): 63 U/L (normal < 55 U/L), and *γ*-glutamyltransferase (*γ*-GT): 176 U/L (normal: 12–64 U/L), so fatty infiltration of the liver was revealed after an abdominal ultrasound. Serum transaminase levels rose remarkably at the age of 12 months (AST: 608 U/L, ALT: 390 U/L, and *γ*-GT: 421 U/L) and have since remained elevated, while his weight and height were below the 3^rd^ percentile and have remained so ever since (Table 1). Hence, an extensive workup was carried out to rule out all possible causes, including metabolic disorders. Organic acid urinary analysis was normal in multiple nonconsecutive samples (including 3-methylglutaconic acid in urine), so a distinct metabolic disease was not detected. In addition, besides the fact that whole exome sequencing (WES) was performed, bioinformatic analyses failed to detect a pathogenic or likely pathogenic molecular defect. Diagnostic liver biopsy, performed at the age of twelve months, revealed severe steatosis with fibrosis (nonalcoholic steatohepatitis) in the pathology study, so ursodeoxycholic acid was initiated at the age of 25 months and *γ*-GT levels were subsequently normalized. In addition, after the age of 3 years, due to the clinical suspicion of mitochondrial dysfunction, a carnitine supplementary diet was initiated.

During infancy, in regular follow-up, he was gradually diagnosed to suffer from multiple disorders. More specifically, he developed microcytic anemia without iron deficiency, (at 12 months of age) and hypercalciuria (at the age of 3 years), and his 25-OH vitamin D levels were in the upper normal limits, with no vitamin D substitution therapy (range: 95–150 ng/ml and normal values: 30–150 ng/ml), along with low 1.25 OH2 vitamin D levels (range: 13.7–15.5 ng/ml and normal values: 19.6–54.3 ng/ml), since the age of 2 years. On the other hand, kidney function was normal, and levels of serum calcium, phosphate, alkaline phosphatase, and parathormone were also normal. Further investigation with a DEXA scan was performed, and the child was diagnosed with osteopenia (lumbar spine *z*-score: −2.9). Brain magnetic resonance imaging (MRI), at the age of 18 months, showed small areas of increased signal symmetrically bilaterally in the posterior part of the pons (MRI findings were unchanged in the follow-up examination at the age of 4 years) (Table 1).

**Figure 1:**
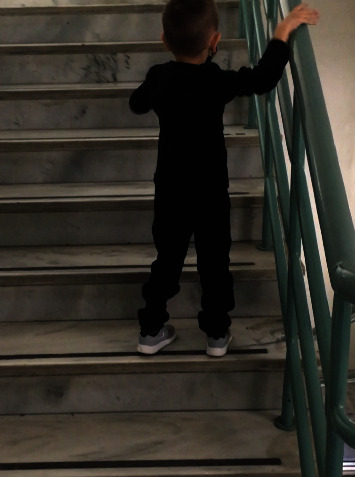
Difficulty in climbing stairs. At the age of five years, the boy walks up the stairs with support, placing two feet on each stair. There is also a wide-based gait (his feet are spaced widely apart).

**Figure 2:**
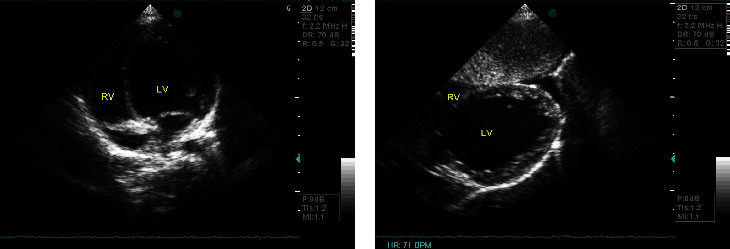
(a) Echocardiography from apical 4-chamber view: dilated left ventricle with global shape. (b) Subcostal view: noncompaction features at the apex. LV: left ventricle and RV: right ventricle.

Then, at his regular follow-up, at the age of five years, a slight delay in conquering psychomotor milestones was noted. So, taking again the patient's medical history, it was discovered that the boy's activity and energy levels were always less than normal for his age. He also had mild ataxia (there was a wide-based gait with recurrent falls and especially difficulty in climbing stairs) (Figure 1). Furthermore, he had a mild delay in speech development, so he was under speech therapy since last year. In addition, an echocardiogram revealed an enlarged left ventricle with global shape and preserved systolic and diastolic function (ejection fraction: 55–58%) (Figure 2(a)). The electrocardiograms showed inverted T waves in precordial leads V5, V6, and QT prolongation (QTc max: 545 ms), but the 24-hour EGC (Holter) did not reveal rhythm disturbances. Therefore, an ACE inhibitor (captopril) and digoxin were initiated, in order to restore cardiac function, along with propranolol for QT prolongation, to reduce the risk of cardiac arrhythmias. Since then, repeated echocardiograms revealed normal left ventricular systolic function, with mild evidence of noncompaction features at the apex (Figure 2(b)). Furthermore, QTc interval was improved (QTc: 482–487 msec) and remained so thereafter.

**Figure 3:**
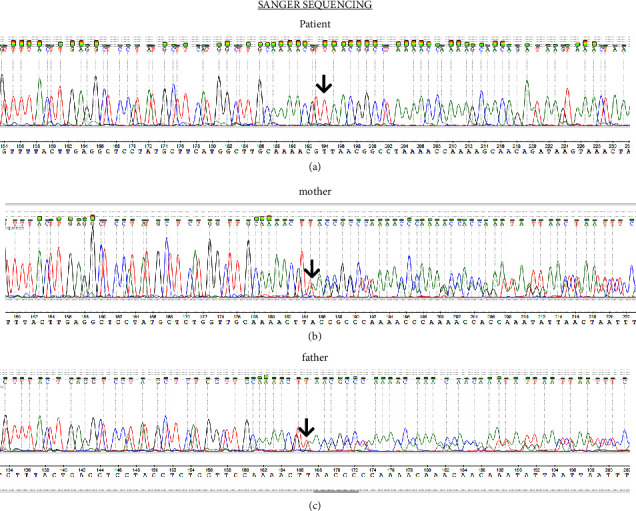
Sanger sequencing (from the patient and parental samples): (a) the homozygous insertion of a single base (T) at codon 21 in exon 3 of the *DNAJC19* gene, c.62dup (p.Tyr21Ter) in the patient's sample (arrow); (b) the heterozygous insertion of a single base (T) at codon 21 in exon 3 of the *DNAJC19* gene, c.62dup (p.Tyr21Ter) in the maternal sample (arrow); (c) the heterozygous insertion of a single base (T) at codon 21 in exon 3 of the *DNAJC19* gene, c.62dup (p.Tyr21Ter) in the paternal sample (arrow).

Taking into consideration all the above findings, a reanalysis of WES was performed, which revealed a homozygous insertion of a single base (T) at codon 21 in exon 3 of the *DNAJC19* gene, c.62dup (p.Tyr21Ter). The presence of the specific variant was confirmed by Sanger sequencing (Figure 3(a)). This variant is reported at a frequency of 0.00000795% in the gnomAD population database, without any homozygotes being reported so far. However, this variant has been previously reported in ClinVar as pathogenic (Variation ID: 1299515) and has been found in a patient exhibiting clinical symptoms compatible with 3-methylglutaconic aciduria, type V (#610198) [9]. Both parents were also checked (with the Sanger method), and they were found to have the same heterozygous p.Tyr21Ter variant (Figures 3(b) and 3(c)). In addition, mutations responsible for congenital long QT syndrome or vitamin D metabolic disorders have not been detected.

So the patient was diagnosed with methylglutaconic aciduria type V, by the identification of a homozygous pathogenic variant (MGCA5#610198) in the *DNAJC19* gene [7].

## 3. Discussion

Our case not only expands the clinical characteristics of MGCA5 disorder but also highlights the necessity and power of genetic analysis for detecting a diagnosis, when the metabolic screen is negative. This sequence change creates a premature translational stop signal (p.Tyr21*∗*) in the *DNAJC19* gene, and it is expected to result in an absent or disrupted protein product. Loss-of-function variants in the *DNAJC19* gene are known to be pathogenic (PMID: 16055927, 27928778, 22797137, and 35611801) and have been previously reported in patients presenting with early onset dilated cardiomyopathy with conduction defects, nonprogressive cerebellar ataxia, testicular dysgenesis, growth failure, and 3‐methylglutaconic aciduria [1, 2, 4, 5] (Table 2).

Clinical features of our patient, such as growth failure, anemia, ataxia, male genital abnormalities, dilated cardiomyopathy, and long QT, were found to be similar to those already described in the Canadian population [1, 10]. However, our patient also suffered from osteopenia and hypercalciuria, and his 25-OH vitamin D levels in repeated measurements were in the upper limits, along with low 1.25 OH2 vitamin D levels. These features have never been described before in other cases [2–4]. As serum calcium levels were normal in all measurements and other known causes of hypercalciuria were excluded, we believe that hypercalciuria could be related to osteopenia. In addition, we assume that disorders of vitamin D levels are presumably related to malnutrition, rather than severe liver involvement (steatohepatitis). Indeed, our patient recently started a nutrition program that has been designed by a clinical dietician: a hypercaloric diet with increased meal frequency, enhanced with medium-chain triglycerides (MCT), that improved his vitamin D levels.

Machiraju et al., in a retrospective study, described the percentages of the main clinical characteristics of the disease. More specifically, from 43 Hutterite patients between 2005 and 2015, 93% developed ataxia, 80% had cerebellar atrophy, 62% had abnormal genitalia, 83% had QT prolongation, and 50% had DCMA [10].

In daily clinical practice, despite the fact that the combination of early-onset cardiomyopathy with multiorgan features raises the possibility of metabolic disease, it is often difficult and time-consuming to obtain a specific etiological diagnosis in children. Exome sequencing in these cases seems to be the gold standard, assuming that MGCA5 disorders are not so rare but underdiagnosed or undiagnosed unless genetic analysis is performed [4].

In addition to the heterogeneity of clinical manifestations, the biochemical markers of mitochondria dysfunction in plasma and urine (which were not elevated in our patient) made the diagnosis of the disease even more difficult. However, it has already been described that 3-methylglutaconic aciduria may be transient in these patients [3].

The initial WES analysis failed to detect the particular variant, as there were not enough phenotypic criteria at that time to interpret the variant as the possible genetic etiology (the patient was referred due to cryptorchidism/microphallus and transaminasemia). When the patient developed new phenotypic data, including dilated cardiomyopathy, ataxia, and mild mental retardation, the reanalysis of WES revealed the pathogenic variant.

In our patient, cardiac involvement was the most characteristic feature, leading to the diagnosis of this rare condition. In a retrospective review of 17 Hutterite patients, echocardiography revealed dilated cardiomyopathy in 13/17, but no one had left ventricular noncompaction findings. Six out of these patients improved with treatment, and two of them had a resolution of cardiomyopathy. As for electrocardiographic abnormalities, QTc prolongation was diagnosed in 8/17 patients (6 with dilated cardiomyopathy and 2 without cardiomyopathy) and nonspecific ST/T changes in 5/17 patients [6].

The medical approach to mitochondrial cardiomyopathies remains challenging, and it is mainly based on drugs used for conventional heart failure therapy (such as ACE inhibitors). However, in MGCA5 patients, Greenway et al. have proved a favorable effect by adding digoxin to ACE inhibitor therapy [11]. More specifically, they noted that the initiation of digoxin was associated not only with stabilization of systolic function but also with a reduction in prolonged QTc interval. The suggested mechanism seems to be a rise in mitochondrial ATP that modifies cardiolipin production in metabolic pathways. However, as they clearly mention, digoxin cannot be sufficient as monotherapy [11]. Therefore, in our patient, digoxin in a maintenance dose was started, along with b-blockers and captopril, in order to reverse left ventricular remodeling.

In summary, genetic analysis, along with the already known clinical characteristics, unmasked the diagnosis. Features, such as vitamin D disorders or anemia, may be related to malnutrition, as they resolve with appropriate diet. Identifying new clinical features in patients may contribute to a better understanding of the underlying pathogenic mechanism, leading to novel therapies. Early identification and treatment will retard the progression of cardiomyopathy and improve survival. As metabolic screening is not always diagnostic, genetic testing, including WES or clinical exome sequencing (CES), is helpful in clinical practice, leading to proper medical management.

## Figures and Tables

**Table 1:** Age-related clinical features and laboratory findings of our patient.

Age	Clinical features	Laboratory findings
Neonatal life	(i) Birth weight: 10^th^ centile (ii) Height: 50^th^ centile (iii) Genital abnormalities: (bilateral cryptorchidism, small hypoplastic scrotum, and extreme microphallus) (iv) Bilateral testicular dysgenesis	(i) Low testosterone levels (ii) Male karyotype (46,XY)

6 months	(i) Failure to thrive (weight < 3^rd^ centile, height: 25^th^ centile)	(i) Increased liver enzymes (ii) Fatty infiltration of the liver (abdominal ultrasound)

12 months	(i) Failure to thrive (weight < 3^rd^ centile, height < 3^rd^ centile)	(i) Remarkably elevated liver enzymes (ii) Steatohepatitis in liver biopsy (iii) Microcytic anemia (without iron deficiency) (iv) Normal organic acid urinary analysis (including 3-methylglutaconic acid)

18 months	(i) Failure to thrive (weight < 3^rd^ centile, height < 3^rd^ centile)	(i) Brain MRI (small areas of increased signal symmetrically bilaterally in the posterior part of the pons)

2 years	(i) Failure to thrive (weight < 3^rd^ centile, height < 3^rd^ centile)	(i) Increased 25(OH)Vit D levels, low 1.25 (OH)2Vit D levels

3-4 years	(i) Failure to thrive (weight < 3^rd^ centile, height < 3^rd^ centile) (ii) Mild delay in speech development	(i) Hypercalciuria

5 years	(i) Delay in conquering psychomotor milestones (ii) Mild ataxia	(i) Dilated cardiomyopathy with preserved ejection fraction (ii) Prolonged QT interval (iii) DEXA: osteopenia

7.5 years (after starting a special diet)	(i) Weight: 3^nd^−15^th^ centile, height: 3^rd^ centile	(i) Normal levels of 25(OH)Vit D and 1.25 (OH)2Vit D (ii) Normal hemoglobin levels

**Table 2:** Clinical and molecular characteristics of patients with 3-methylglutaconic aciduria type V, including this report.

Study features	Davey 2006	Ojala 2012	Al Teneiji 2016	Ucar 2017	Al Tuwaijri 2022	Our patient
No. of patients	18 hutterite patients	2 Finish brothers	1 patient	1 Turkish patient	1 Arabic patient	1 Greek patient

Age at diagnosis	N/A	N/A	13 years old	2 months	3 years old	5.5 years old

Clinical features at the disease onset	(i) DCMP (11/18) (ii) Growth failure (iii) Ataxia (10/18) (iv) Genital anomalies	(i) DCMP (ii) Growth retardation (iii) Ataxia (iv) Cryptorchidism (1/2)	(i) DCMP (transient) (ii) Ataxia (iii) Hypotonia	(i) DCMP noncompaction (ii) Growth failure (iii) Cryptorchidism (iv) Severe neurodevelopmental delay	(i) DCMP (ii) Developmental delay	(i) Genital anomalies (ii) Growth retardation (iii) Transaminasemia (iv) Anemia

Additional features	(i) Long QT (6/18) (ii) Transaminasemia (8/18) (iii) Hepatic steatosis (5/18) (iv) Anemia (12/18) (v) Mild MR (10/18) (vi) Optic atrophy (4/18) (vii) 3-MGA	(i) Noncompaction CMP (ii) Long QT (iii) Anemia (iv) Hypotonia	(i) 3-MGA (transient) (ii) Developmental delay (iii) Mild transaminasemia	(i) Sensorineural hearing loss (ii) Bilateral basal ganglia lesions (iii) Dysmorphic facial features (iv) Anemia (v) Transaminasemia (vi) 3-MGA	(i) Growth retardation (ii) Hypotonia	(i) DCMP with noncompaction features (ii) Prolonged QT (iii) Liver steatosis (iv) Vit D disorders (v) Hypercalciuria (vi) Osteopenia

Genetic analysis	Homozygous splice site mutation (IVS3-1G > C)	Homozygous polymorphism c.285A ⟶ C (p.Gly95 = )	Homozygous five base pair splice site deletion in the *DNAJC19* (c.280þ1_280þ 5delGTAAG)	Variant c.63delC (p.Tyr21*∗*) in homozygous state	Single homozygous frameshift at codon 54 in exon 4 (c.159del) [Phe54Leufs*∗*5]	Homozygous insertion of a single base (T) at codon 21 in exon 3 of the *DNAJC19* gene, c.62dup (p.Tyr21Ter)

N/A: not available; DCMP: dilated cardiomyopathy; 3-MGA: 3-methylglutaconic aciduria; MR: mental retardation.

## Data Availability

Data that support this case are included within the article.

## Supplementary Materials

Supplementary Materials Methodology information (including data analysis and PCR technique) is described in the supplementary files.
