# Supplementary material for: Genital Abnormalities and Growth Retardation as Early Signs of Dilated Cardiomyopathy with Ataxia Syndrome
Source: Case Rep Genet. 2024 Jan 20;2024:8860889. doi: 10.1155/2024/8860889 (PMC10821800; doi:10.1155/2024/8860889)
Supplement: Supplementary Materials — Methodology information (including data analysis and PCR technique) is described in the supplementary files. [file 8860889.f1.pdf]

METHOLOGY INFORMATION

Information about clinical exome:

Data analysis was performed using the Varsome Clinical Version 11.8 (Saphetor) bioinformatic analysis platform. At the time of interpretation and analysis the variant was not reported in the ClinVar, Uniprot and Decipher databases and it was not reported in patients in the scientific literature. It was reported at the Genome Aggregation Database (gnomAD) database. At present, there is a ClinVar entry (Variation ID: 1299515) and LOVD entry: https://databases.lovd.nl/shared/variants/0000881289#00006571 classifying the variant as pathogenic.

***Information about the technique*:**

The PCR reactions were performed in a 50 µl reaction mixture containing 1X Go Taq®Reaction Buffer, 1.5mM $MgCl_2$, 0,2mM each dNTPs and 1U Go Taq® DNA polymerase (Promega, USA) using specific primers 5'-TCTGATGTTTCAGGGTAGCACCA-3' (Forward) and 5'-TGAGAAAGGGTGGGAATTAGAGAAG-3' (Reverse). ~100ng of extracted DNA was used as template. The cycling conditions consisted of an initial denaturation step at 95◦C for 5 min, followed by 35 cycles of 30 sec at 95◦C, 30 sec at 60◦C, and 30 sec at 72◦C, with a final cycle of 5 min at 72◦C. Direct sequencing was performed with the BigDye Terminator cycle sequencing kit v3.1 (Applied Biosystems, Foster City, CA) and analyzed by capillary electrophoresis using an ABI Prism 3130*xl* (Applied Biosystems). Unipro UGENE v.1.31.1 software was used to compare the experimental sequences to the reference one (NM_145261).
